# Synthetic image dataset of shaft junctions inside wind turbines in presence or absence of oil leaks

**DOI:** 10.1016/j.dib.2021.107538

**Published:** 2021-11-03

**Authors:** Matteo Cardoni, Danilo Pau, Laura Falaschetti, Claudio Turchetti, Marco Lattuada

**Affiliations:** a STMicroelectronics, via C. Olivetti 2, Agrate Brianza, I-20864, Italy; b Department of Information Engineering, Università Politecnica delle Marche, Ancona, Via Brecce Bianche 12, Ancona I-60131, Italy; c STMicroelectronics, via Tolomeo 1, Cornaredo, I-20010, Italy

**Keywords:** Oil leaks, Wind turbines, Anomaly detection, Machine learning, Image dataset, Image classification

## Abstract

This paper presents a dataset of images generated via 3D graphics rendering. The dataset is composed by pictures of the junction between the high-speed shaft and the external bracket of the power generator inside a wind turbine cabin, in presence and absence of oil leaks. Oil leak occurrence is an anomaly that can verify in a zone of interest of the junction. Since the wind turbines industry is becoming more and more important, turbines maintenance is growing in importance accordingly. In this context a dataset, as we propose, can be used, for example, to design machine learning algorithms for predictive maintenance. The renderings have been produced, from various framings and various leaks shapes and colors, using the rendering engine Keyshot9. Subsequent preprocessing has been performed with Matlab, including images grayscale conversion and image binarization. Finally, data augmentation has been implemented in Python, and it can be easily extended/customized for realizing any further processing. The Matlab and Python source codes are also provided. To the authors’ knowledge, there are no other public available datasets on this topic.

## Specifications Table

**Table:** 

Subject	Industrial Engineering
Specific subject area	Components inside wind turbines
Type of data	Images,
	Matlab source code for preprocessing and Python source codes for data augmentation
How data were acquired	3D graphics rendering,
	preprocessing in Matlab (source code is included),
	data augmentation in Python (source codes are included)
Data format	Digital images (*.jpg and *.png format);
	Matlab script (*.m format);
	Google Colaboratory notebooks (*.ipynb format).
Parameters for data collection	16 camera positions (field of view fixed at 39.6${}^{\circ }$), metallic texture (not painted steel and white painted steel), 20 leaks shapes, colors of each leak, light conditions, saturation (normal, enhanced), images representation (RGB, grayscale, binary), spatial transformations (horizontal flip, vertical flip, rotation, width shift, height shift,shear range, zoom), noise addition.
Description of data collection	Original images have been generated by 3D graphics rendering. The features defined in the rendering are: camera position, metallic texture, leak shapes, leak colors. Light condition is always diffuse.
	Matlab preprocessing: light condition alteration (from natural to warm, cold and low intensity), saturation enhancing, grayscale conversion and binarization of the grayscale images.
	Python data augmentation: erosion, horizontal flip, vertical flip, rotation, width shift, height shift, shear range, zoom, noise addition.
Data source location	Institution: Università Politecnica delle Marche, Department of Information Engineering, via Brecce Bianche, 12
	City/Town/Region: Ancona (AN)
	Country: Italy
	Latitude and longitude for collected samples/data: 43${}^{\circ }$35’12.9”N 13${}^{\circ }$31’00.5”E
Data accessibility	Repository name: Oil leak dataset
	data: https://doi.org/10.17632/nbxzxn3ffk.1
	Direct URL to data: https://data.mendeley.com/datasets/nbxzxn3ffk/1

## Value of the Data

• This set of images was produced by a synthetic generation process by using a 3D graphics software renderer. This avoided an annoying, very challenging, and costly task of capturing them live into the wind turbine. The virtual rendering of the scenes indeed was the only viable choice available to us because the other alternative could be to acquire picture in the generator cabin of the wind turbine. Unfortunately this is only accessible to certified operators (and not certainly to the authors of this paper) under strict operative conditions which do not leave space for picture shooting to anybody else. With such methodology we modelized and virtually operated different kind of sensors (e.g. rolling shutter, global shutter), lens aperture (e.g. 39.6 degree), point of views, pixel defectivity, noise at different signal to noise ratio and including for example thermal noise e other kinds. Moreover by morphing key frames, motion picture sequences were derived to mimic the oil leak expansion over the time.
• Anyone working in the field of image processing can use it for various purposes such as anomaly detection for wind turbines, image segmentation, motion detection, image coding, pixel labelling, etc.
• The dataset can be used to design, for example, an oil leak detection algorithm.Expensiveness and riskiness of wind turbine maintenance can be simulated using a virtual reality system of cameras acquiring images to test any kind of algorithm.
  Oil leaks detection in wind turbines is a challenging problem, since it can provoke fires inside the turbine and because human prompt intervene is very difficult to happen, being both dangerous and very expensive [1], [2].
• Machine learning approaches can be adopted for the definition of the actual condition of the system [3], [4], [5] including the capability to detect anomalies [6], [7], [8], [9], avoiding the riskiness of wind turbine maintenance.
• The source code used for preprocessing, data augmentation, and noise addition can be edited in order to apply different transformations, thus increasing the data diversity.

## 1 Data Description

The main folder Oil_leak_dataset contains the sub-foder Junction_images, where are located 22 directories of images, named Group_1 to Group_22, and the Matlab preprocessing script, named Preprocessing.m. Groups from 1 to 20 contain images with leaks, each one containing images with a different leak shape, while groups 21 and 22 contain images without leak.

The main folder Oil_leak_dataset also contains two Jupiter notebooks for the generation of the grayscale images (Oil_leak_grayscale_augmentation.ipynb) and of the binary images (Oil_leak_binary_augmentation.ipynb). Both the grayscale and the binary images are obtained with the preprocessing script starting from RGB images.

The directory tree is shown below:



### 1.1 Images with oil leak

Group folders from 1 to 20 include images with leaks. The organization of the images with oil leak inside the directory Junction_images, before applying Matlab preprocessing, is the following:



The images in each folder are named as follows:

g<group number>s<set number>.<image number><label>.<extension>.

With this notation, g specifies the Group and s specifies for set. After the point there are the image number and a label which describes the type of preprocessing applied to the image. Since these images have not been preprocessed, the nat (i.e., natural light) is used.

In each group the leak shape is the same while it is worth noting that in the whole dataset the images with the same <image number> are generated with the same framing. Each set, from set_1 to set_6, contains 16 RGB images, specifically:

- set_1: Not painted steel texture, brown leak.
- set_2: Not painted steel texture, green leak.
- set_3: Not painted steel texture, gray leak.
- set_4: White painted steel texture, brown leak.
- set_5: White painted steel texture, green leak.
- set_6: White painted steel texture, gray leak.

**Fig. 1:**
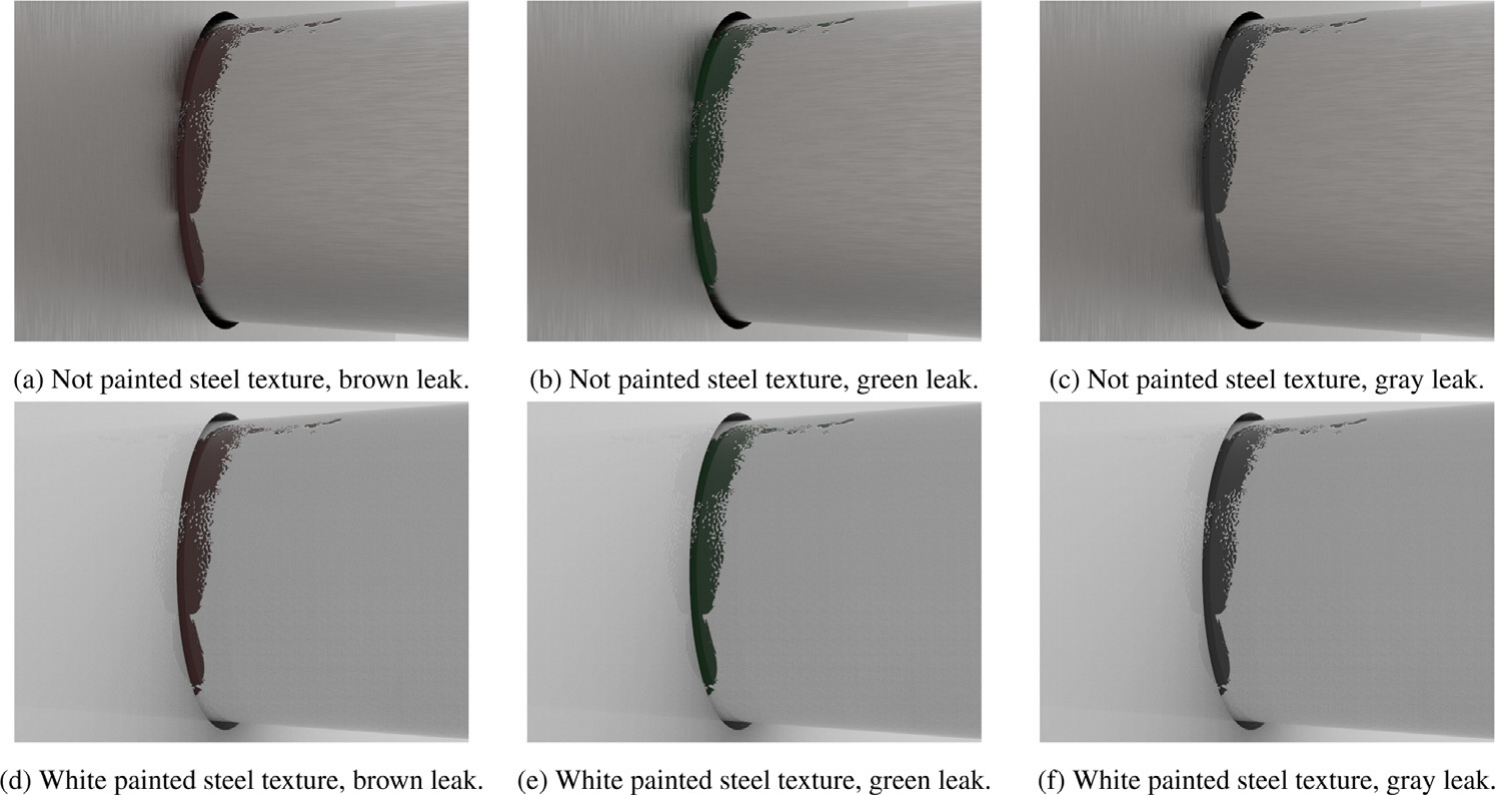
Natural light (i.e., not preprocessed) images from group 4, one for each set. Each image has the same <image number> in the sets and as a consequence they have the same framing.

Fig. 1 shows examples of images with the same framing (i.e., with the same <image number>) of Group_4, from all the 6 sets (g4s<set number 1-6>.2nat.jpg). In particular, Fig. 1a–c refer to not painted steel texture with brown leak, green leak and gray leak respectively, while Fig. 1d–f refer to white painted steel texture with brown leak, green leak and gray leak respectively.

**Fig. 2:**
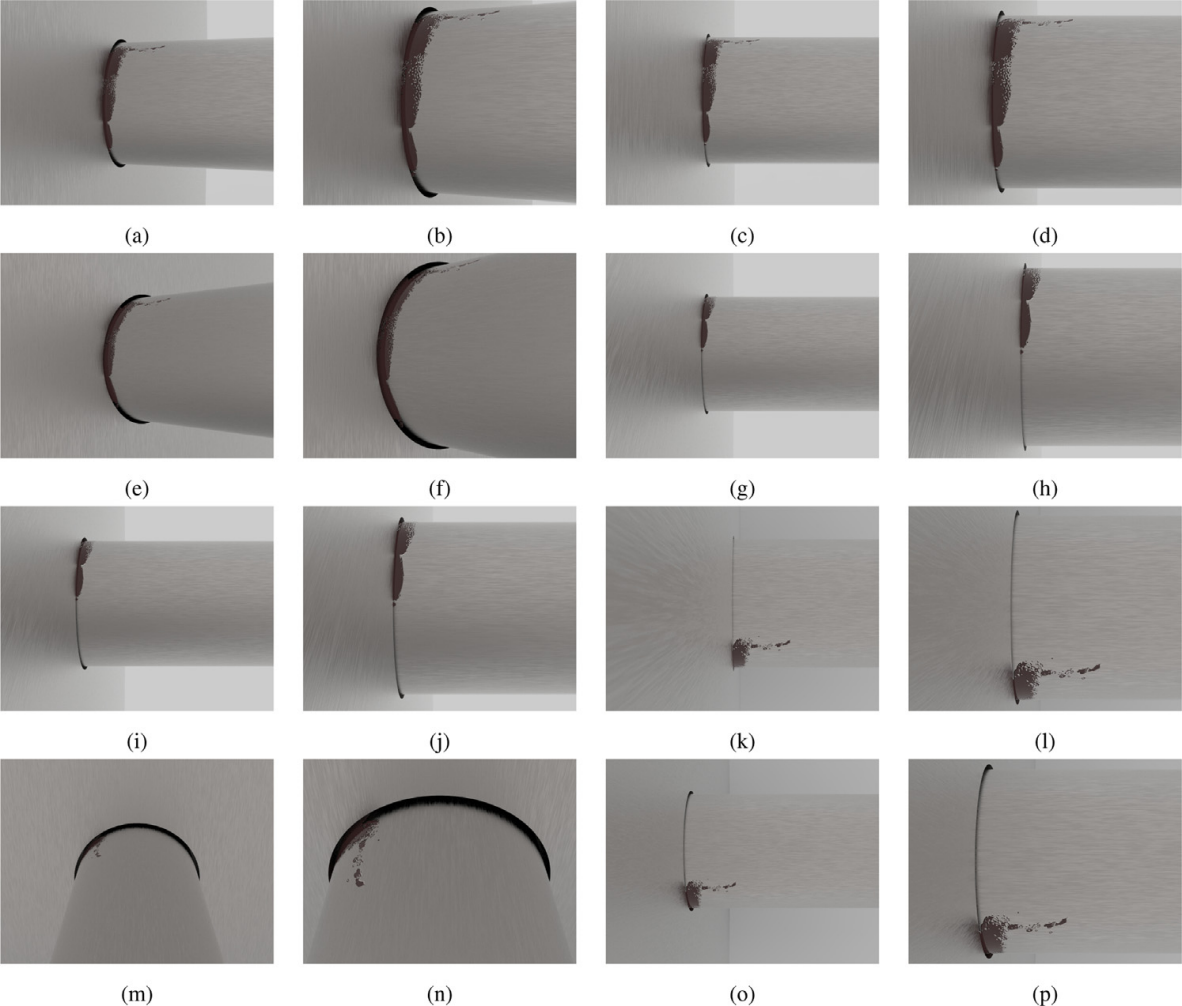
All 16 images contained in the directory Group_1/set_1/natural_light.

Figure 2 shows the images inside the directory Group_4/set_1/natural_light

(g4s1.<image number 1-16>nat.jpg). Particularly, Fig. 2a, b, d, e, f show the left view of the natural light images, Fig. 2g–j depict the bottom view and Fig. 2k–p report the top view. Because of the symmetrical nature of the problem it has been decided not to render images from the right side of the junction.

### 1.2 Images without oil leak

**Fig. 3:**
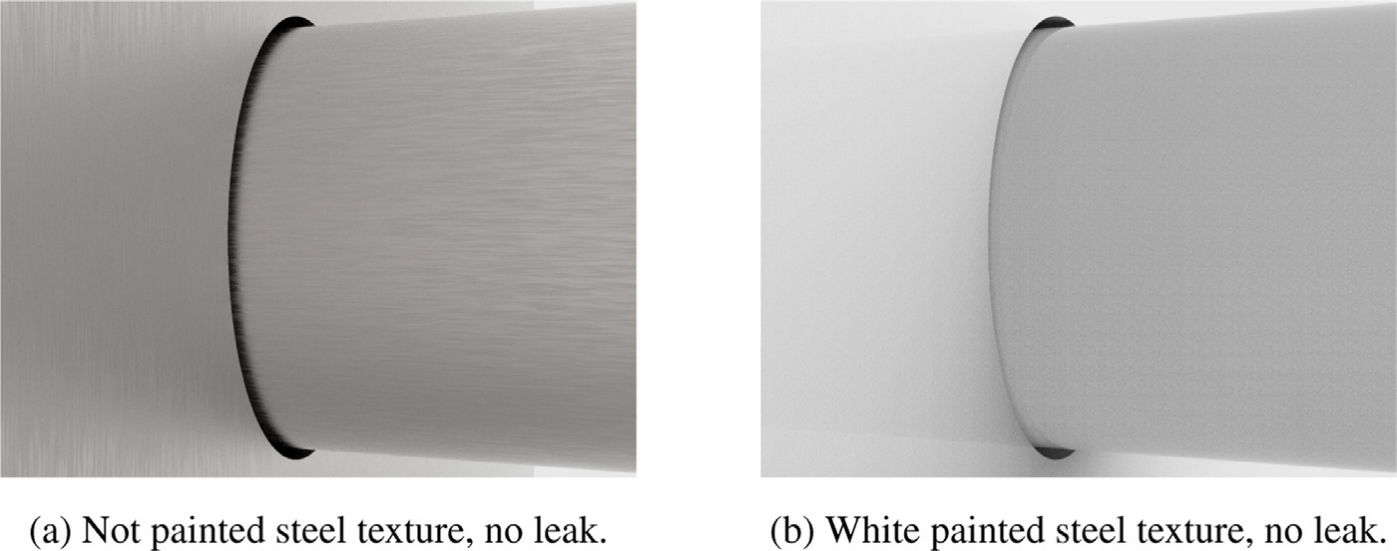
Natural light (i.e. not preprocessed) images without leak.

Groups 21 and 22 include images without oil leaks. Group_21 contains images rendered with not painted metallic texture, while Group_22 contains images rendered with white paint texture. The directory tree of the images without oil leak, before applying preprocessing, is the following:



There is only one set per group (in both cases named set_1). Except for the number of directories, the images positions and numbers are the same of the images presenting oil leaks. Fig. 3 shows examples of images not presenting oil leaks, with not painted steel texture (Fig. 3a) and with white painted steel texture (Fig. 3b).

### 1.3 Images quantity

The number of images obtained through rendering is reported in Table 1. The number of images obtained after the preprocessing (described in Section 3.3) is reported in Table 2. Finally, the number of images obtained through binary and grayscale data augmentation (described in Section 3.4), applying all the possible transformations, is reported in Table 3.

**Table 1:** Number of images obtained through rendering.

	RGB	Grayscale	Binary
With leak	1,920	-	-
Without leak	32	-	-

**Table 2:** Number of images after preprocessing.

	RGB	Grayscale	Binary
With leak	11,520	640	640
Without leak	192	32	32

**Table 3:** Number of images after data augmentation.

	RGB	Grayscale	Binary
With leak	11,520	20,480	30,720
Without leak	192	1,024	1,536

## 2 Experimental Design, Materials and Methods

### 2.1 3D model

**Fig. 4:**
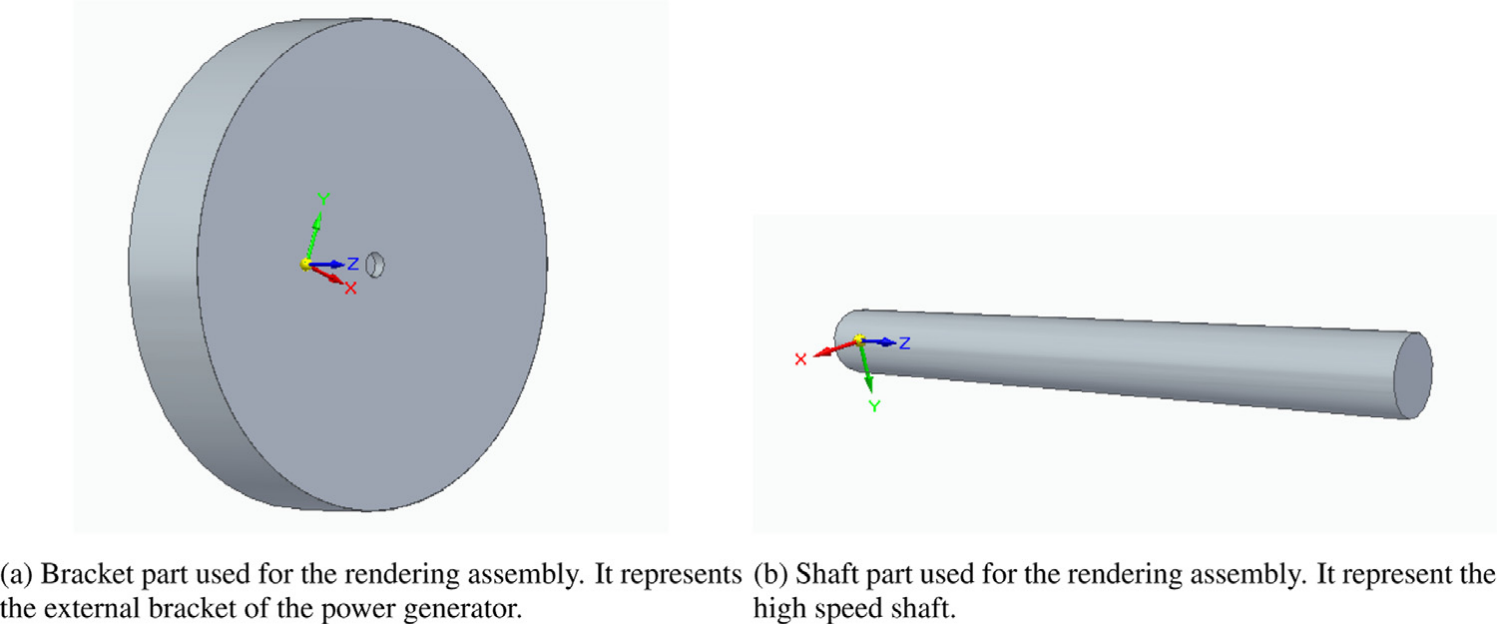
Parts used to compose the assembly used as rendering basis.

**Fig. 5:**
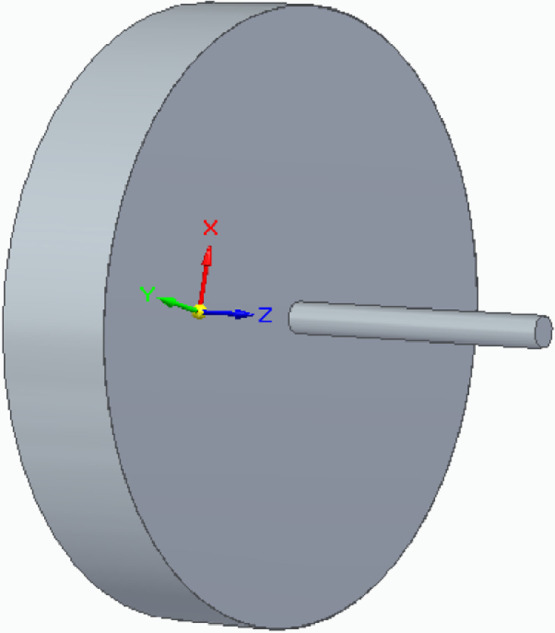
Assembly composed of the parts in Fig. 4.

The starting point of the renderings has been two 3D parts generated in SolidEdge 2020^1^, shown in Fig. 4. The part in Fig. 4a (bracket part) is a cylinder with an internal cylindrical cavity. The cylinder has a diameter of 2,000 mm and a height of 400 mm and the cavity hole has a diameter of 105 mm and a depth of 50 mm. The part in Fig. 4b (axis part) is also cylindrical, with a diameter of 100 mm and a length of 1,000 mm. The two parts compose an assembly, shown in Fig. 5. The axis part is placed inside the cavity of the bracket part. The diameters of the axis part and of the cavity in the bracket part are not the same: they are respectively 100 mm and 105 mm. This has been done in order to simulate some space in their coupling, as can be seen in the figures in Section 2.

### 2.2 Rendering

#### 2.2.1 Leaks images

**Fig. 6:**
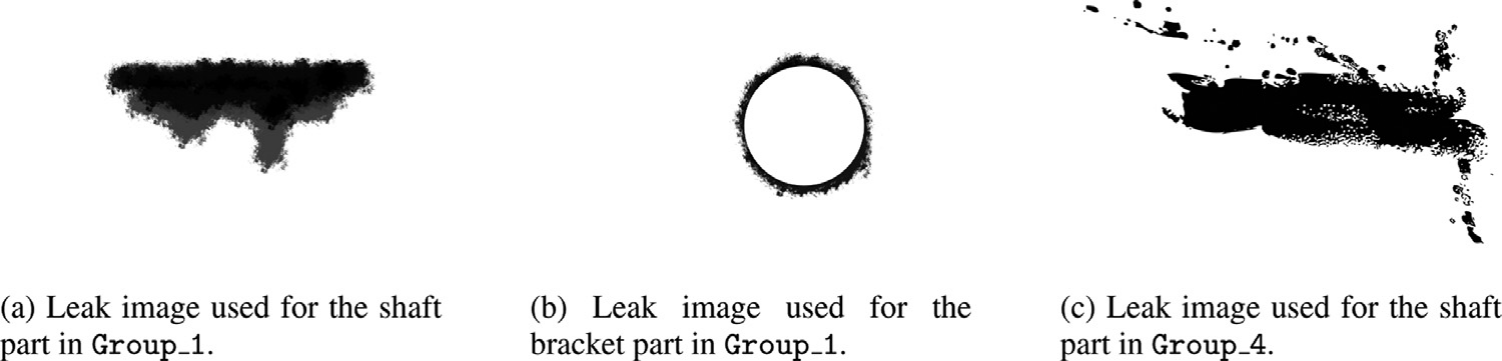
Leaks images examples. All the leak images have been produced as white png images with transparent background. Here they are represented in black for the sake of visibility.

Leak images have been produced using the image manipulation software Gimp^2^. These images have been produced in png format in order to generate them with a transparent background. Brush tools of white color have been used to produce the leak images. One or more leaks have been produced for each Group folder for images with leak. Only in some cases leak images have been generated to be positioned not only on the shaft part but also on the bracket part of Fig. 4a. In Fig. 6 examples of leak images are shown, with the leaks colored in black for the sake of visibility. Specifically, Fig. 6a and b refer to the leaks, used for Group_1, which are positioned, respectively, on the shaft part and on the bracket part of the model, while Fig. 6c shows the leak of Group_4 which has been positioned on the shaft part.

The generated leak images have been imported in KeyShot9^3^ as textures. They have been changed in color, positioned, resized, and wrapped (for the leaks to be used for the axis part of Fig. 4b). The leak textures have also been elaborated with bump mapping and displacement mapping to let them to appear three-dimensional in the renderings.

#### 2.2.2 Light, background and virtual cameras specifics

The environment has been chosen as a completely white background, spreading uniform and diffuse white light. The brightness level has been tuned to make the background of a light gray color, giving the images a more natural look.

The 3D model has been imported into Keyshot9 and the renderings have been performed using 16 virtual cameras with an aperture of 39.6${}^{\circ }$. The camera positions have been kept fixed for the generation of all the images.

#### 2.2.3 Rendering specifics

The rendering has been performed using an Intel i5-6200U (dual core, clock frequency 2.3 GHz) with the following following specifics:

• **Samples**: Images have been rendered with 4 samples (i.e., calculated 4 times in order to increase accuracy) [10].
• **Ray bounces**: The number of ray bounces, that is the number of times the light reflexes are calculated, have been set to 6 (that is the default value).
• **Pixel blur**, i.e., the amount of blur added to avoid over-sharp images, has been disabled in order to keep sharp shapes.
• **Anti-aliasing**, which is used to smooth jagged edges, has been set to 1, as indicated as sufficient in [10].
• **Shadow quality** has been kept to the minimum (i.e., 1) in order to reduce the rendering times, since in [10] is reported the dramatic increase of time due to shadow quality raising.
• **Resolution**: All the images obtained with rendering have a resolution of 1024×768 pixels.

### 2.3 Matlab preprocessing

The Matlab preprocessing script can been applied to the rendered images. It has been used with Matlab version the R2020b and requires the Image Processing Toolbox [11]. The folder structure of the images including oil leaks, after the application of the preprocessing script, is the following:



For the images without leak, after applying the preprocessing, the directory tree is the following:



#### 2.3.1 Preprocessed RGB images

The images obtained from the RGB preprocessing are in jpg format. To each image of the folder natural_light, inside each set, 5 different RGB preprocessings are applied, each one identified by a label as explained in Section 2.2. The labels and the corresponding preprocessing are:

• low1: luminance value decrease: 0.5 times the original.
• low2: luminance value decrease: 0.3 times the original.
• sat: saturation enhancement of 5 times the original.
• cold: modification of light temperature to warmer: +5 points in red, -15 points in blue.
• warm: modification of light temperature to colder: -15 points in red, +5 points in blue.

**Fig. 7:**
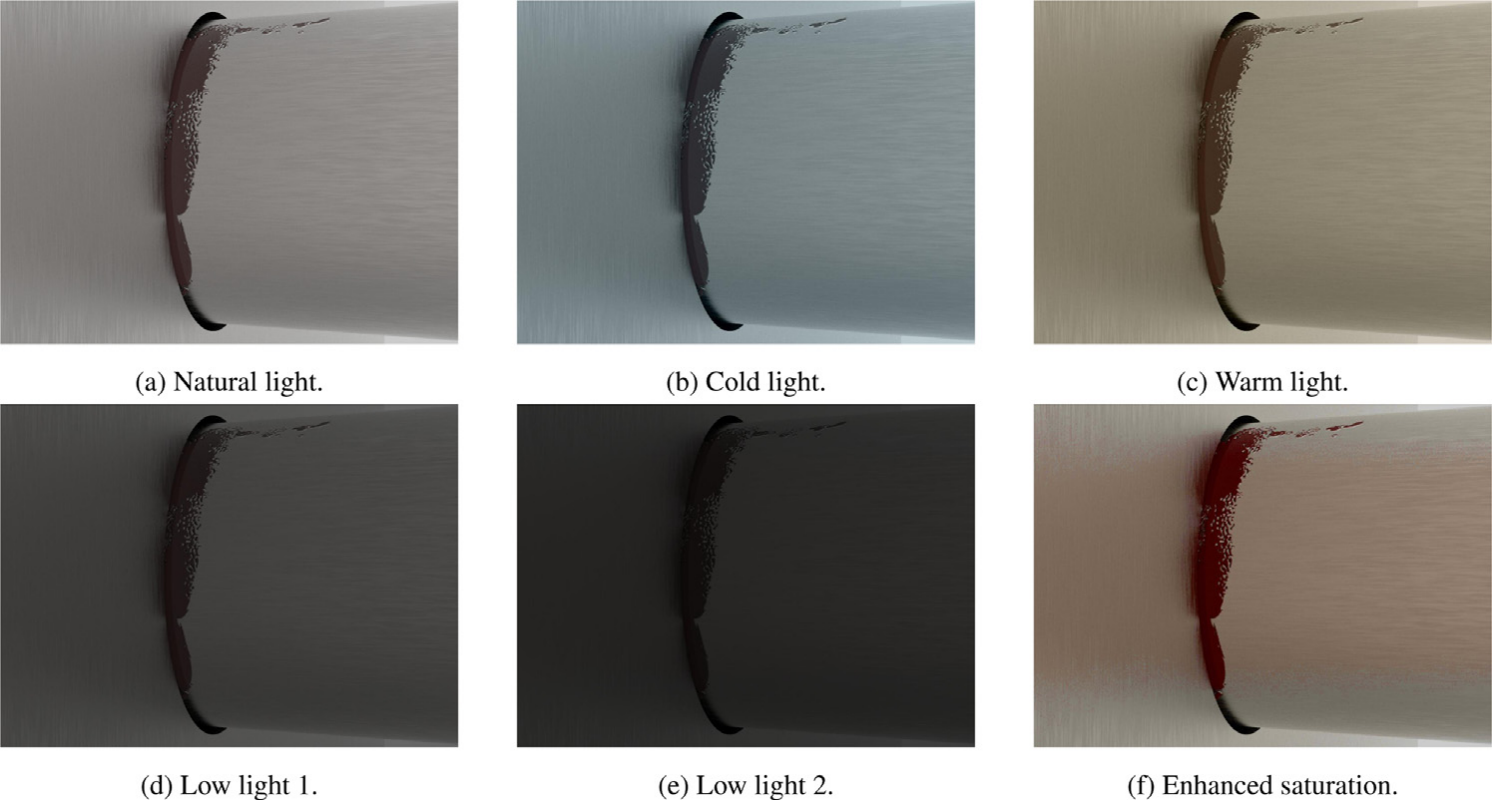
Application of different preprocessing to an image of Group_4.

Fig. 7 shows examples of the same RGB image (g4s1.2nat.jpg - not painted steel texture and brown leak) when different light conditions are applied: natural light (Fig. 7a), cold light (Fig. 7b), warm light (Fig. 7c), low light 1 (Fig. 7d), low light 2 (Fig. 7e) and enhanced saturation (Fig. 7f).

#### 2.3.2 Grayscale images

The grayscale images are in jpeg format and characterized by the gs label (Section 2.2). The conversion from RGB format to grayscale eliminates hue and saturation, retaining the luminance value of the image.

The grayscale images in the directories grayscale_1 are obtained from the set_1 images of each group, while the images in grayscale_2 are obtained from the set_4 images of the same group. This is why the <set_number> of the images in the grayscale_2 folders is 4.

**Fig. 8:**
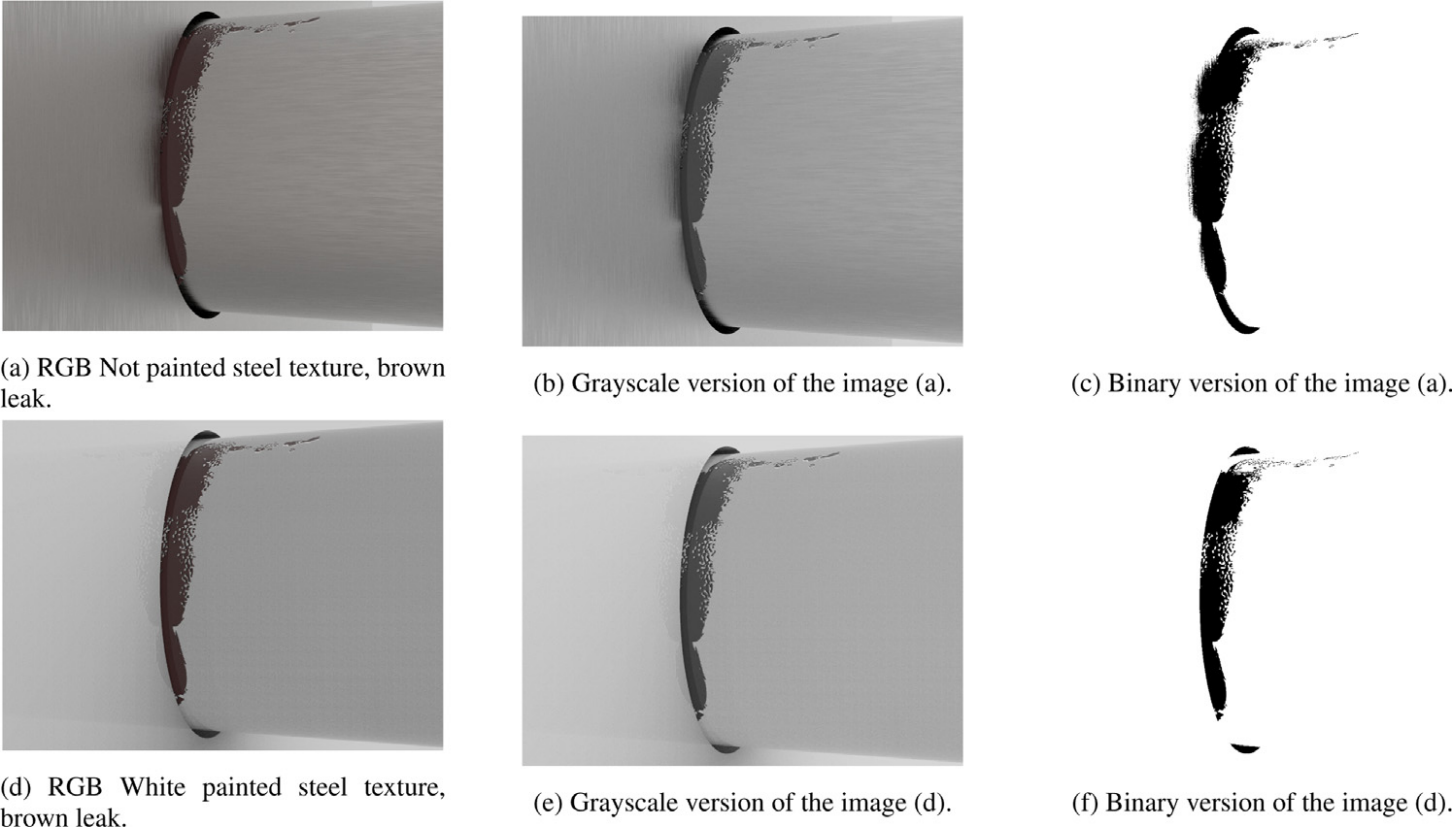
Natural light images from set_1 and set_4 (a and d) converted to grayscale (b and e) and binary (c and f).

For Group_21 and Group_22 (i.e., images without leak) there is only one set of images (Section 2.2). For this reason only one directory of grayscale images has been generated, named grayscale_1, as shown in the directory tree for images without oil leaks in Section 3.3. Fig. 8 shows an example of the process of grayscale conversion (and subsequent binarization, that will be explained in Section 3.3.3). Starting from Fig. 8a, Fig. 8b is first obtained and finally Fig. 8c. Fig. 8d–f show the same process.

#### 2.3.3 Binary images

The binary images format is png and the associated label is bin (Section 2.2). The binary images have been saved in png format because jpg format does not allow to have images only composed of 0 and 1 values. The binarization of the grayscale images is performed calculating a threshold using the Otsu’s method [12].

The images in the directories binary_1 are obtained with from the grayscale_1 directories, while the images in binary_2 are obtained from the directory grayscale_2. Since the images from the folders grayscale_2 are obtained from set_4 of the respective group, the images <set_number> is 4.

As explained in Section 3.3.2 Group_21 and Group_22 will contain, after preprocessing, only one grayscale images directory, named grayscale_1. For this reason these two groups, after preprocessing, will contain only one binary images folder, named binary_1. Fig. 8 shows an example of the process of grayscale conversion and binarization.

#### 2.3.4 Preprocessing code explaination

First, the name of the subfolders are set for the levels while the top level is the dataset folder where the Group directories can be found.



Next, the values for the preprocessing are set.



After that, for every directory containing the ”Group” string, the level 2 string is used as name for the created directories. Directories to contain grayscale and binary images are calso reated. In this section it can be seen how for the directories of images without leak (i.e., Group_21 and Group_22) only one directory for grayscale and one directory for binary are created (as explained in Sections 3.3.2 and 3.3.3).



The same process is reiterated for the set directories, saving them in level 3. This is implemented nesting a second for loop inside the first one.



A level 4 subdirectory natural_light is created in each set of each group by a third for loop is nested in the second.



Every time the a natural_light directory is reached, all the images contained in it are elaborated through a fourth for loop, nested in the 3rd. The following code details the preprocessing for grayscale and binary conversion (only for the directories set_1 and set_4), and the saving in the Group directories.



After the conversion of the natural light images into grayscale and binary, the original images are processed as reported in Section 3.3.1. This is implemented by the following code.



### 2.4 Python data augmentation

Data Augmentation is a process to enhance the data quantity and quality, producing useful variability for training more accurate deep neural networks [13].

Scripts for augmentation are provided for grayscale and binary images, since the number of images of these classes are smaller than RGB images generated after preprocessing. The data augmentation scripts have been designed with Python 3.7.11 and use the ImageDataGenerator class, provided by the Keras (version 2.5.0) library [14] and other functions from Tensorflow (version 2.5.0) and scikit-image (version 0.16.2).

Both the augmentation scripts perform the following operations:

1. Download the corresponding images directory (binary or grayscale).
2. Import necessary libraries (e.g., Numpy, Tensorflow, Scikit-Image, os, Pathlib).
3. Enable/disable the augmentation functions commenting/uncommenting from transformation names lists.
4. Define the augmentation functions that are not methods of the ImageDataGenerator class (using Tensorflow and scikit-image).
5. Apply the augmentation functions.
6. Visualize the augmented images, compared to the original ones (this step is optional).
7. Save the augmented data. The dataset can be saved to the user’s Google Drive.

The transformations that are performed are reported in Table 4.

**Table 4:** Table of transformations applied to binary and grayscale images and the corresponding labels added to the augmented images.

	Grayscale	Binary	Corresponding label
Erosion filter	x	✓	erosion
Rotation	✓	✓	rotation
Horizontal flip	✓	✓	horizontalFlip
Vertical flip	✓	✓	verticalFlip
Height shift	✓	✓	heightShift
Width shift	✓	✓	widthShift
Zoom	✓	✓	zoom
Shear range	✓	✓	shearRange
Salt and pepper noise 1%	✓	✓	s&p1%
Salt and pepper noise 5%	✓	✓	s&p5%
Gaussian noise	✓	x	gauss

The default specifications of the aforementioned transformations are: erosion with disk filter of radius 5, rotation range angle of 40${}^{\circ }$ (with nearest fill mode), width shift range of 0.2 (with nearest fill mode), height shift range of 0.2 (with nearest fill mode), zoom range of 0.2, shear range of 0.2, salt&pepper noise at 1% and 5% percentages, gaussian noise with mean 0 and variance 0.01. These default values can modified by the user.

**Fig. 9:**
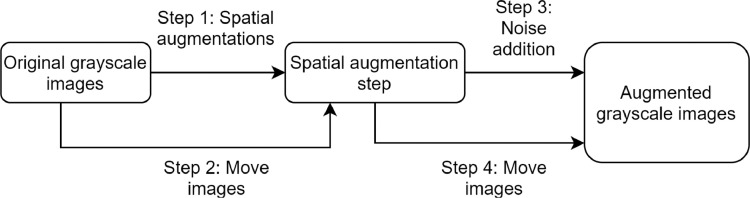
Diagram of the augmentations pipeline for grayscale images. In this way the noise addidion is performed both to the spatially augmented images and to the original ones.

The augmentation process for grayscale images has been designed to add noise both to the original images and the spatially augmented ones (scheme in Fig. 9).

**Fig. 10:**
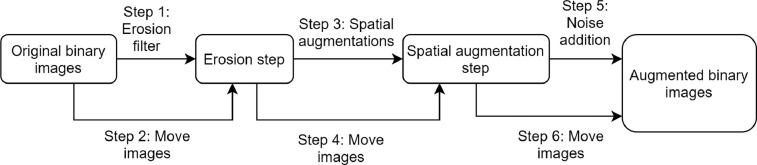
Diagram of the augmentations pipeline for binary images. The spatial augmentations are performed after the erosion filter applied both to the eroded and to not eroded images. All these images are subsequently processed for saòt&pepper noise addition.

As for the binary images, the augmentation has been designed in order to apply the erosion filter to the original images, subsequently apply the spatial augmentations to the eroded and original images and finally apply the noise addition to all these images combinations. The scheme in Fig. 10 synthesizes the pipeline.

For both the scripts the original directory structure is preserved: when an augmentation is applied, a label is added to the image name, preceded by and underscore. The labels corresponding to the transformations are reported in Table 4.

#### 2.4.1 Custom data augmentation functions

Here the augmentation functions that use the scikit-image library (version 0.16.2) are reported. The first reported function is the grayscale salt&pepper noise addition with 1% noise level. In this code it can be seen that the images from the ImageDataGenerator class have an extra dimension that must be removed before the noise addition and added again after the elaboration. Furthermore, the images from the ImageDataGenerator class are represented by single precision floating point values and must be converted in 8-bit unsigned integers (*uint8*) before applying the noise addition.



The other functions for noise addition are not reported since they are very similar to the just presented one. Binary s&p noise addition does not need the conversion to *uint8* representation.

The following function apply the erosion filter for binary images, using scikit-image (version 0.16.2). Also in this function the extra dimension in the image must be discarded before the erosion and re-added after it.



#### 2.4.2 Data augmentation instructions for binary images

In order to be augmented, binary images need to be separated from the grayscale and RGB images. The images directory tree can be the same obtained from the preprocessing. These directory needs to be uploaded in zip file in the user’s Google Drive. After that, in the script Oil_leak_binary_augmentation.ipynb, in the code after STEP 1 - Dataset download, the variable dataset_name needs to be updated with the binary images directory name (this variable is originally instantiated with ”Oil_leak_binary”). The shareable link of the zipped directory needs to be retrieved, setting the visibility to ”Anyone with the link”, and this link must be assigned as string (i.e., with quotation marks) to the variable dataset_url. Finally, the link’s file ID (between the second-last and the last slash characters) must be inserted at the end of the string assigned to the variable dataset_url4wget.

#### 2.4.3 Data augmentation instructions for grayscale images

Grayscale images need to be separated from the binary and RGB images. The same rules of the binary data augmentations (Subsection 3.4.2) have to be applied. The script that has to be used to augment grayscale images is Oil_leak_grayscale_augmentation.

## Ethics Statement

The work did not involve any human or animal subjects, nor data from social media platforms.

## CRediT authorship contribution statement

**Matteo Cardoni:** Conceptualization, Methodology, Software, Validation, Formal analysis, Investigation, Data curation, Writing – original draft, Writing – review & editing, Visualization. **Danilo Pau:** Conceptualization, Methodology, Investigation, Data curation, Writing – original draft, Writing – review & editing, Visualization, Supervision, Project administration. **Laura Falaschetti:** Conceptualization, Methodology, Software, Validation, Formal analysis, Investigation, Writing – original draft, Writing – review & editing, Visualization. **Claudio Turchetti:** Conceptualization, Methodology, Investigation, Writing – review & editing, Visualization. **Marco Lattuada:** Conceptualization, Methodology, Investigation, Writing – review & editing, Visualization.

## Declaration of Competing Interest

The authors declare that they have no known competing financial interests or personal relationships which have, or could be perceived to have, influenced the work reported in this article.

## Footnotes

1 https://solidedge.siemens.com/en/.

2 https://www.gimp.org/.

3 https://www.keyshot.com/.
